# Progress in Diabetes

**Published:** 1904-01-02

**Authors:** 


### Progress in Diabetes.

*Concluded from page* 228.

A case of bronzed diabetes was reported by G. Parker[11] at the Swansea meeting. The liver and pancreas were shown stained a bright blue from the presence of iron pigment after treatment with acid and ferrocyanide, and the microscopic sections proved that nearly all the tissues of the body were loaded with the same iron pigment. The patient had been a man aged 65, who had enjoyed good health till moderate diabetes appeared four months before his death. The sugar amounted to about $5\frac{1}{2}$ per cent. and the urine to 50 oz. The skin became of a dusky metallic tint, which was most marked on the cheeks, neck, and on the legs, especially around an old varicose ulcer. This greyish brown pigment shaded off into normally coloured skin. He finally died of diabetic coma. The liver and pancreas showed marked hypertrophic cirrhosis, the lymphatic glands were masses of pigment and of a bright chocolate on section, while the deep bronze of the liver and other organs was very striking. The mucous membranes and the intestines on exposure to the air turned to a metallic leaden colour. The pathology has been much disputed both in the French and German schools, but many authorities are now agreed that the primary lesion is a cirrhosis of the liver and pancreas, and that when this has reached a certain stage diabetes follows. The pigment is clearly derived from the blood, but it differs from ordinary hæmoglobin in giving the reactions of free iron. The ordinary means of pigment elimination seem to be destroyed. Another case of bronzed diabetes is given by J. M. Beattie in the *Journal of Pathology and Bacteriology* with references to the literature. *Lipœmia* in diabetes has been occasionally observed since 1799, and a remarkable case is reported by Sir Thomas Fraser[12] where the percentage of fat in the blood reached 16 to 19 per cent. The fat was in the form of an extremely fine emulsion capable of not only passing through the capillaries, but of transuding into the serous cavities; and as its melting point was only 77° it would not tend to form thrombi during life. Fat-globules were, however, seen in films of the blood. The blood at the post-mortem was of a dirty pink, and the vessels were everywhere plugged with a whitish semi-solid matter. There was some leucocytosis, but the blood was otherwise normal. The mode of production of this fat is unknown. It appears to be one of the forerunners of coma, and like the latter may be accompanied with a diminution of sugar in the urine. Fraser inclines to the view that it is due to a modification of the change of the sugar into oxybutyric acid, which is the more usual process in cases tending to coma, and he would treat it by the profuse administration of alkalies as soon as the blood films show the presence of fat-granules.

Hale White[13] records another case and remarks on the pale salmon colour of the retinal arteries and veins, and the granular precipitate in the blood films. This proved to be cholesterine and some proteid carried down with it. There were no oil globules, and indeed no true fat. No connection with the diet could be made out, and as the general condition improved the blood became clearer.

In discussing the varieties of diabetes Rose Bradford[14] points out that diabetes may exist and prove fatal though no sugar appears in the urine, and that five species of diabetes with glycosuria are known. These are (1) that due to digestive disturbances where the sugar depends on the amount of carbohydrate eaten; (2) that due to hepatic failure; (3) that due to pancreatic disease; (4) a renal form probably exists, as shown by the results of phloridzin poisoning; and (5) there is possibly a form due to primary tissue disintegration. Lesions of the nervous system are often credited with a sixth form, but he considers that they only produce glycosuria. Von Noorden[15] has employed as a diet for diabetes a soup containing oatmeal 8 oz, butter 9 oz, and meat 3 oz. This forms a ration for one day, and a certain number of severe cases are said to improve steadily upon this diet, and even to maintain their condition afterwards if the return to ordinary food is made slowly and gradually. Others, however, relapse when the ordinary diet is resumed. Mild forms of diabetes do not frequently give successful results when placed on this "oat cure."

[11] Brit. Med. Jour., Oct. 31. [12] Scottish Med. and Surg. Jour., Sept. [13] Lancet, Oct. 10 [14] Med. Rec., July 4. [15] Ibid., Oct. 3.

